# Supplementary material for: Identification of SH2 Domain-Containing Protein 3C as a Novel, Putative Interactor of Dipeptidyl Peptidase 3
Source: Int J Mol Sci. 2023 Sep 16;24(18):14178. doi: 10.3390/ijms241814178 (PMC10532290; doi:10.3390/ijms241814178)
Supplement: Supplementary file 1 [file ijms-24-14178-s001.zip › ijms-2600010-SI.pdf]

Supplementary material

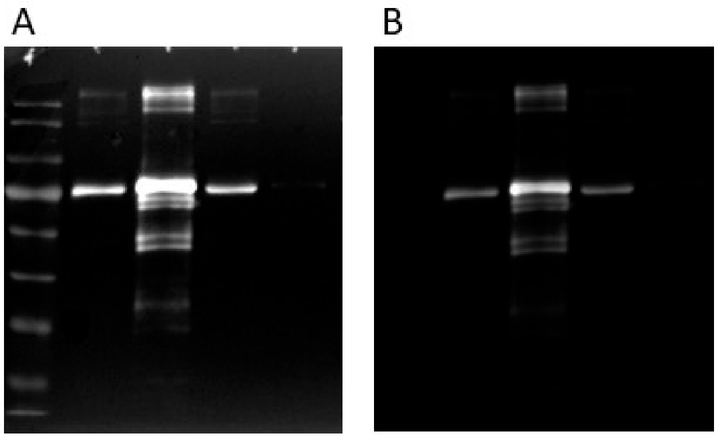

Figure S1. Western blot analysis of the co-immunoprecipitation of HA-DPP3 and KEAP1 (A: merged chemiluminescence and visible; B: chemiluminescence)

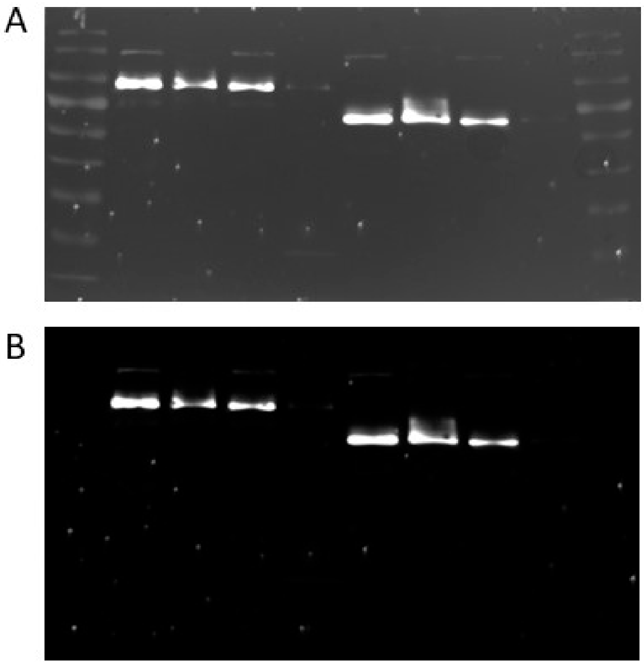

Figure S2. Western blot analysis of the co-immunoprecipitation of HA-DPP3 and SH2D3C-isoform 2 and 3, respectively (A: merged chemiluminescence and visible; B: chemiluminescence)

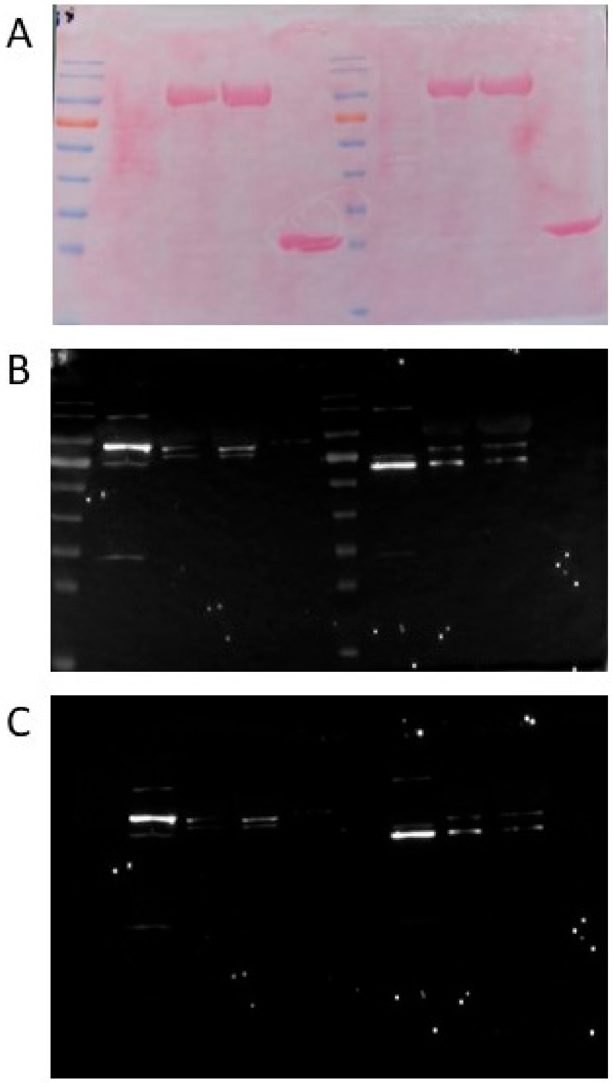

Figure S3. Western blot analysis of the GST-DPP3, GST-DPP3-E451A and GST pulldown of SH2D3C-isoform 2 and 3 (A: Ponceau staining; B: merged chemiluminescence and visible; C: chemiluminescence)

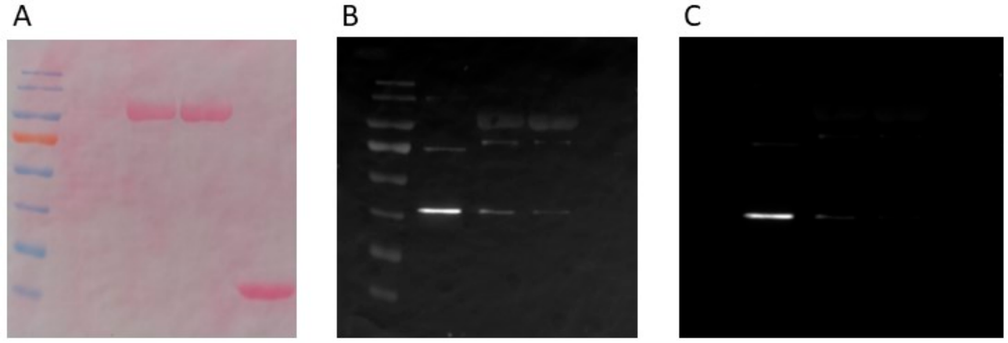

Figure S4. Western blot analysis of the GST-DPP3, GST-DPP3-E451A and GST pulldown of SH2D3C-fragment539 (A: Ponceau staining; B: merged chemiluminescence and visible; C: chemiluminescence)

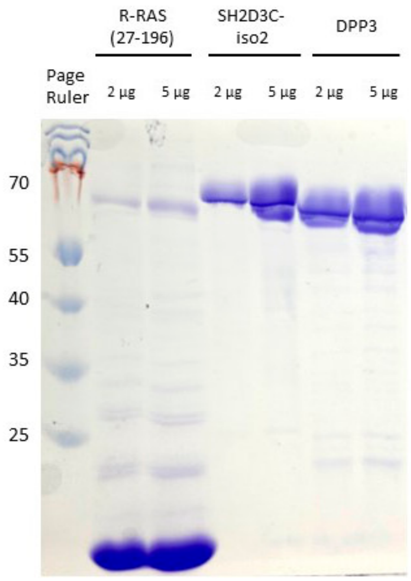

Figure S5. (A) SDS-PAGE (14 %) analysis of purified proteins (RRAS_27-196, DPP3 and SH2D3C-isoform 2) used in GTPase-Glo assay

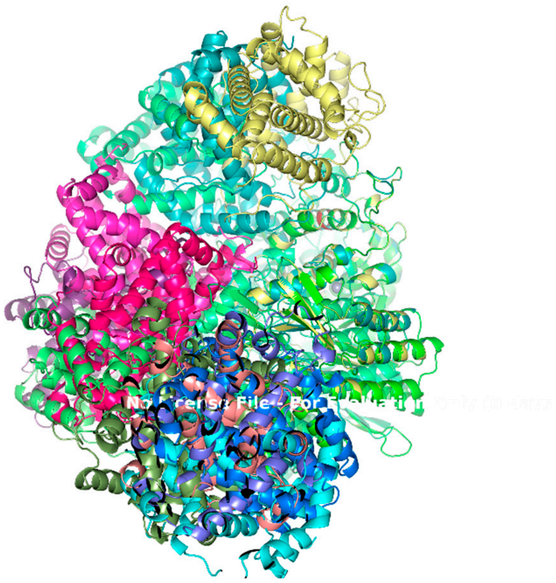

Figure S6. The overlap of the best rated protein-protein docking results

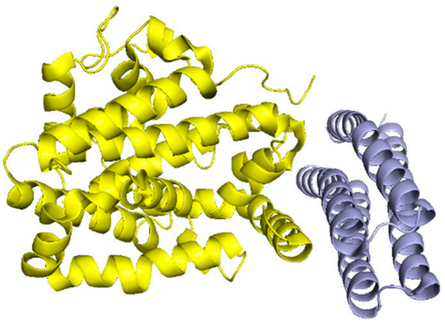

Figure S7. The SH2D3C (NSP3) - p130cas complex crytalographically determined (PDB_id 3t6g). SH2D3C is shown as a yellow and p130cas as a light blue ribon [1]

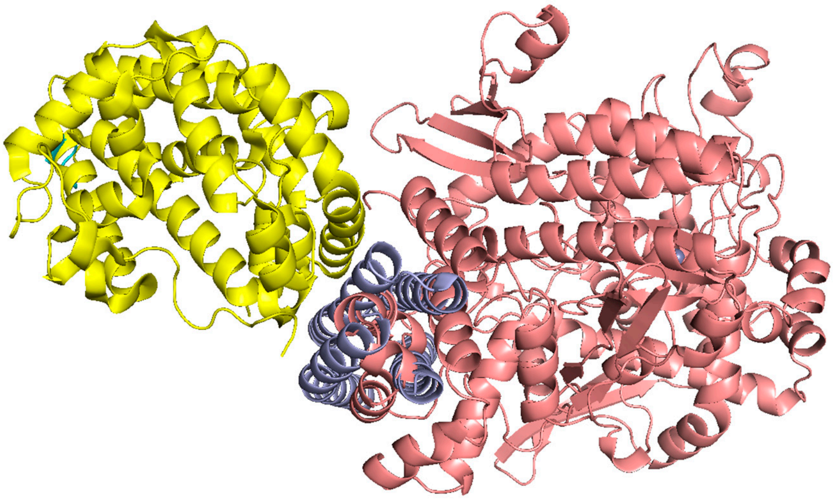

Figure S8. Superposition of SH2D3C (NSP3) in the structure of the crystallographically determined SH2D3C (NSP3) - p130cas complex with SH2D3C in the DPP3 - SH2D3C complexes obtained by docking (model 1, M0). The proteins are shown as ribbons: SH2D3C as yellow, p130cas light blue and DPP3 salmon colored. The zinc ion in DPP3 is shown as a gray sphere.

Table S1. MMGBSA energies calculated for 500 conformers sampled at 10 ns long intervals throughout MD simulations.

Time interval (ns)	Model 1 (**1**)	Model 2 (**2**)	Model 3 (**3**)	Model 4 (**4**)	Model 5 (**5**)
0-10	-2.0498	23.2582	9.5154	-22.254	-27.3818
10-20	-5.0435	14.45	9.6281	-17.8317	-31.7587
20-30	0.6335	12.2781	1.3759	-21.3882	-38.9774
30-40	-11.097	12.2034	0.5333	-14.3276	-40.1269
40-50	-11.2153	15.7201	-12.3807	-11.818	-40.813
50-60	-5.6636	17.602	-16.8353	-11.2413	-32.5318
60-70	-1.6439	15.3425	-19.3385	-2.2865	-35.2631

70-80	-1.386	29.2586	-14.2056	-11.228	-26.7547
80-90	-2.0153	14.45	-14.6175	-20.9924	-40.7935
90-100	-3.2732	12.2781	-17.0513	-21.0878	-38.9552
100-110	-9.2582	26.9854	-27.0466	-18.1174	-32.3653
110-120	-8.1107	17.046	-22.1286	-21.4595	-43.1726
120-130	-6.7439	29.6434	-29.6322	-16.8136	-40.5071
130-140	-4.2571	26.0841	-36.649	-18.1095	-43.5816
140-150	-5.6741	22.8608	-27.9583	-17.8753	-45.0682
150-160	-5.9943	12.2733	-33.6704	-20.2326	-41.1932
160-170	-11.7643	19.6722	-33.8538	-21.2962	-40.6299
170-180	-11.3386	21.3452	-36.8656	-21.6677	-43.5313
180-190	-13.4375	20.8883	-44.9649	-26.074	-35.11
190-200	-12.5538	14.982	-42.0554	-20.4976	-38.1427
200-210	-11.8413		-44.505	-22.1455	-40.3147
210-220	-3.142		-40.6565	-20.08	-20.3388
220-230	-2.3555		-34.0882	-14.513	-24.6556
230-240	-0.91		-41.1824	-14.9297	-28.0908
240-250	-0.3086		-37.8306	-13.0386	-45.8491
250-260	-9.6274		-39.9784	-14.0938	-49.5577
260-270	-15.5859		-35.1981	-19.9653	-43.5163
270-280	-16.0029		-30.6349	-18.4103	-40.7954
280-290	-21.1063		-34.4849	-18.8073	-35.4224
290-300	-20.2185		-24.3995	-14.0615	-36.0945
300-310	-24.8655		-25.6969	-13.0386	-43.7292
310-320	-25.4961		-19.389	-14.0938	-34.2367
320-330	-17.9655		-23.5039	-19.9653	-39.3828
330-340	-17.04		-34.7389	-18.4103	-43.0145
340-350	-14.5702		-30.6646	-15.4013	-51.3042
350-360	-15.9529		-39.9768	-19.9893	-52.8275
360-370	-20.6169		-29.0111	-25.3731	-55.7425
370-380	-21.7599		-29.8122	-29.2021	-49.7883
380-390	-19.0952		-29.2567	-25.7938	-54.0721
390-400	-15.0029		-32.9277	-28.4657	-52.1238
400-410			-33.747	-30.8233	-55.7425
410-420			-29.9035	-29.216	-49.7883
420-430			-26.3494	-26.8955	-54.0721
430-440			-29.78	-28.599	-52.1238
440-450			-29.9502	-25.6187	-51.6892
450-460			-24.8036	-31.0549	-61.0251
460-470			-18.1017	-17.523	-57.094
470-480			-19.2055	-17.1078	-55.873
480-490			-19.4906	-14.8691	-52.9436
490-500			-23.1147	-11.2513	-58.2924
500-510			-23.0992	-8.1348	-72.6836
510-520			-24.9644	-5.413	-70.968
520-530			-25.5451	-5.082	-65.7155
530-540			-34.9402	-2.0726	-69.4991
540-550			-31.2142	-9.0125	-61.5509
550-560			-22.6877	-5.1784	-64.7045
560-570			-40.9062	-5.5555	-68.9686
570-580			-44.6727	-1.0839	-64.1109
580-590			-37.966	-9.2651	-61.4557
590-600			-46.2046	-11.6927	-83.9936
600-610			-37.3573	-6.9671	-74.2161

610-620			-31.5457	-5.4675	-69.4272
620-630			-51.0995	4.443	-59.0799
630-640			-57.4708	4.6024	-60.1609
640-650			-56.2606	1.1906	-60.2755
650-660			-49.2503	-3.4329	-54.6282
660-670			-51.9054	1.1117	-57.1206
670-680			-49.6809	-5.185	-53.2531
680-690			-50.0366	-15.1494	-60.2048
690-700			-48.0331	-5.6104	-58.4852
**Average**	**-10.6**	**19.3**	**-30.5**	**-15.0**	**-49.2**
Av100	-19.2	21.2	-48.3	-26.0	-60.7

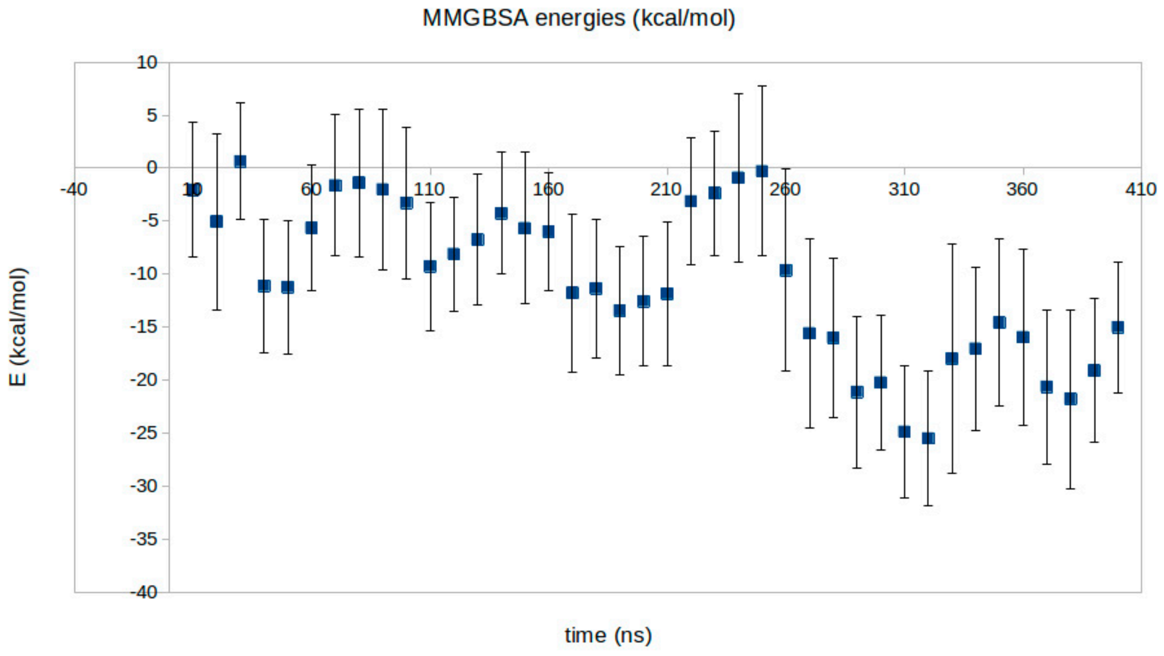
MMGBSA energies (kcal/mol)
E (kcal/mol)
time (ns)

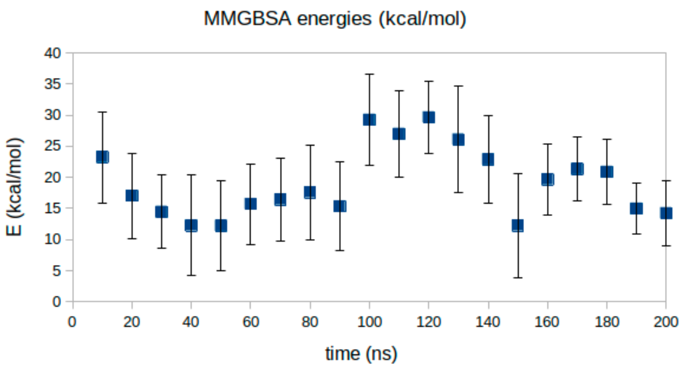
MMGBSA energies (kcal/mol)
E (kcal/mol)
time (ns)

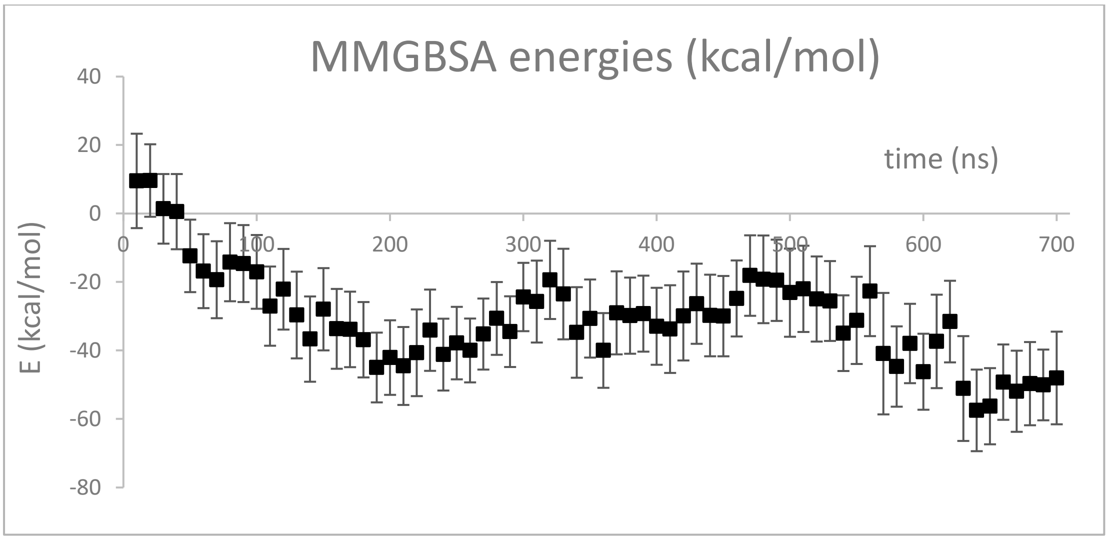
MMGBSA energies (kcal/mol)
E (kcal/mol)
time (ns)

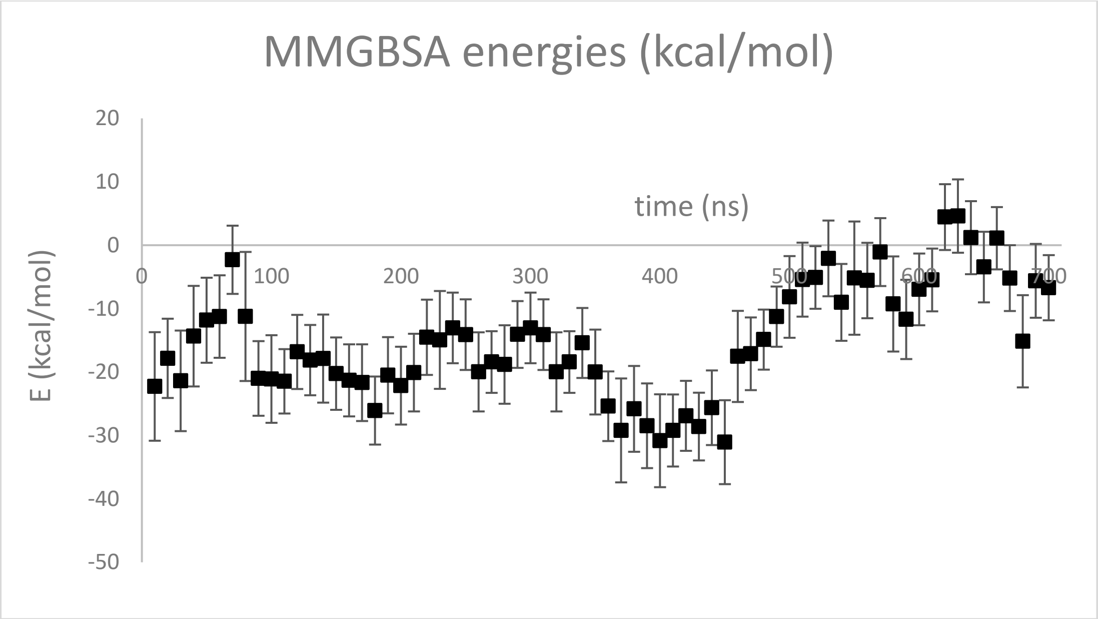

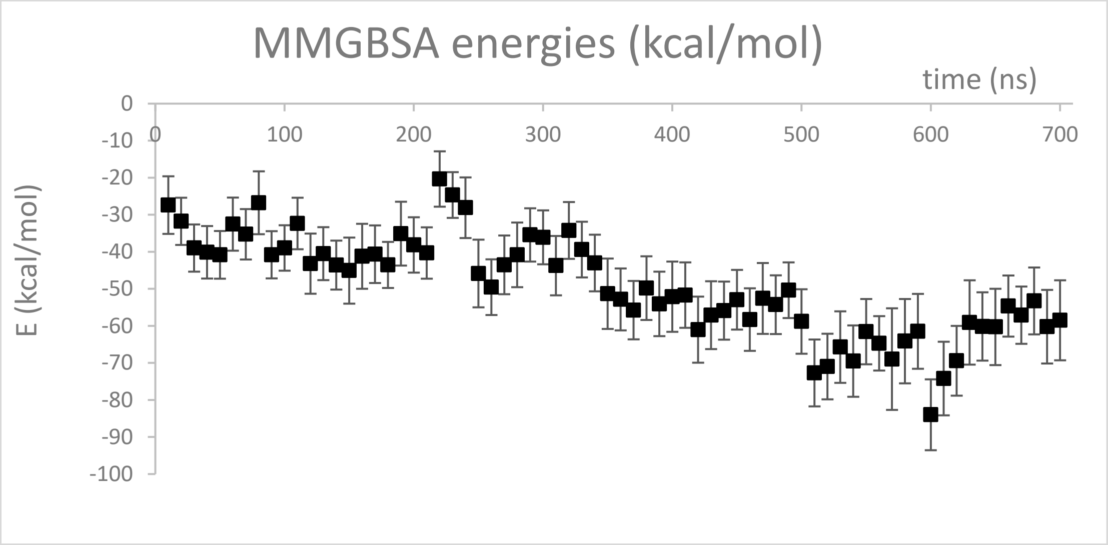

Figure S9. MMGBSA energies (and their standard deviation) for models 1 to 5 (from top to bottom). Values were calculated for conformers sampled at 10-ns intervals throughout MD simulations.

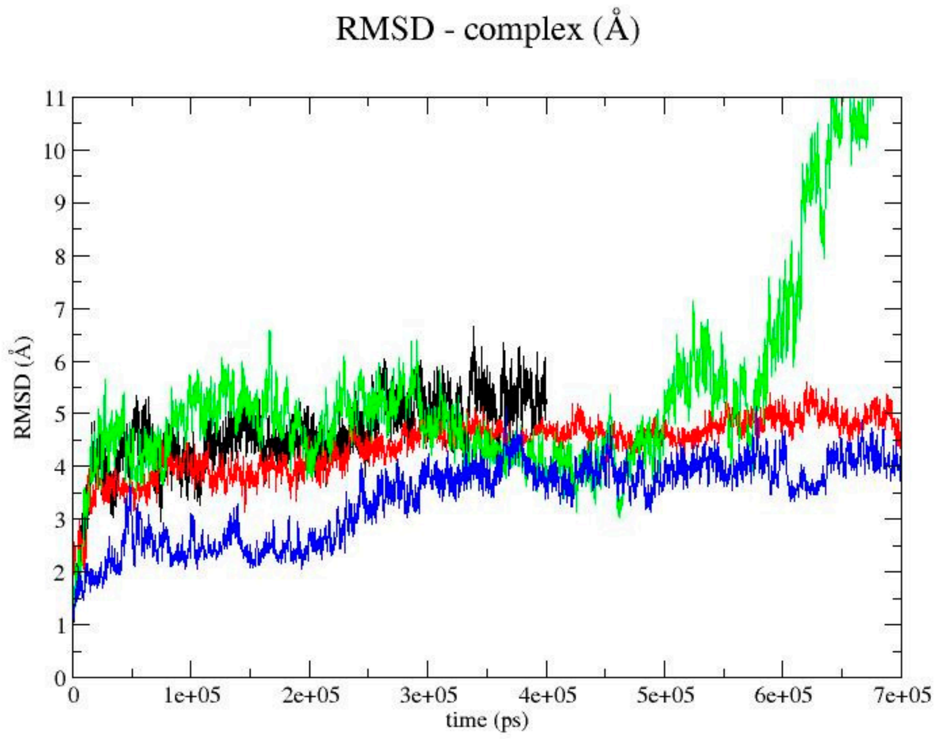

Figure S10. RMSD of the models (DPP3 – SH2D3C complex) during MD simulations (model 1 black, model 3 red, model 4 green, model 5 blue line).

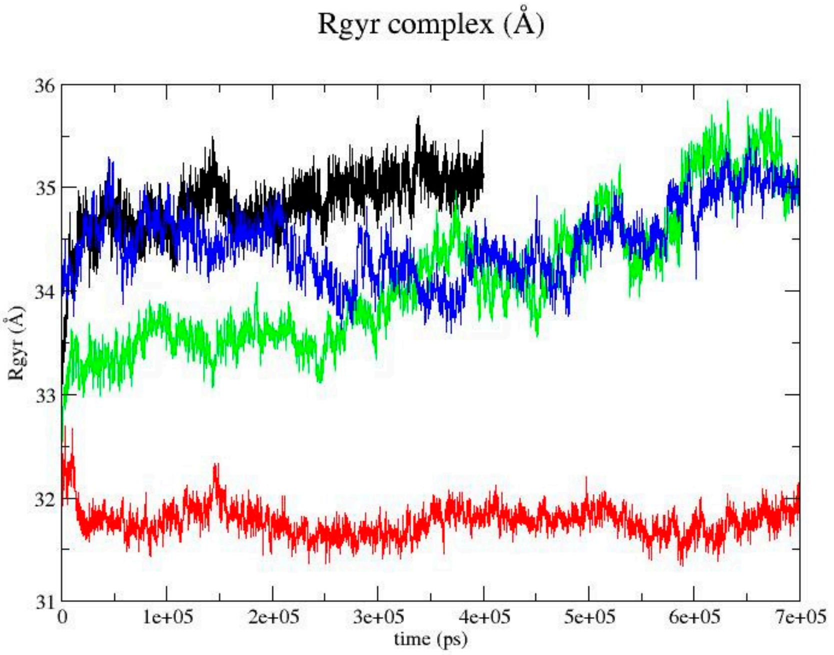

Figure S11. Rgyr of the models (DPP3 – SH2D3C complex) during MD simulations (model 1 black, model 3 red, model 4 green, model 5 blue line).

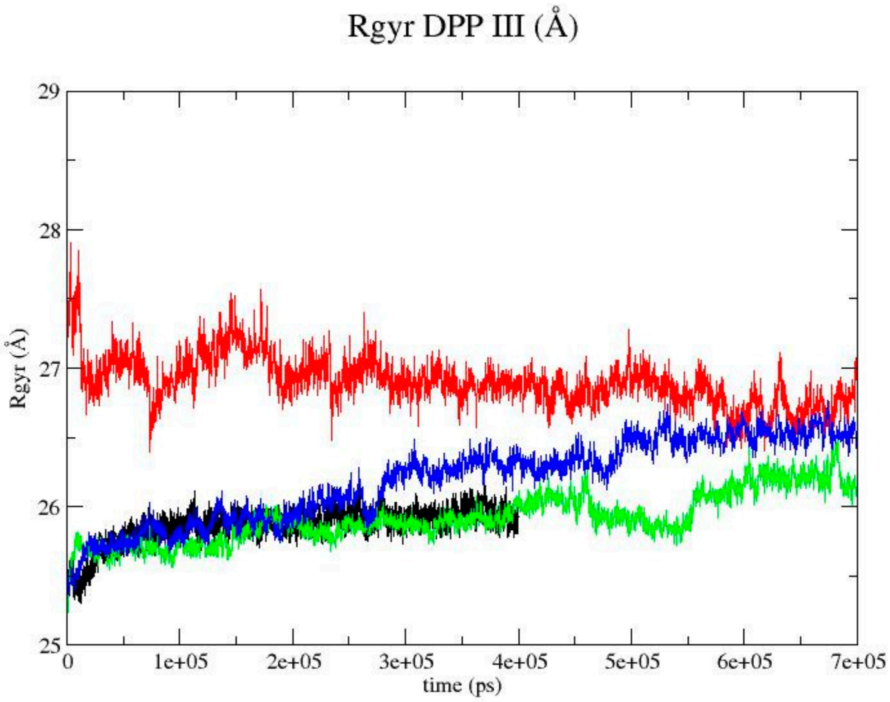

Figure S12. Rgyr of DPP3 in different models (DPP3 – SH2D3C complex) during MD simulations (model 1 black, model 3 red, model 4 green, model 5 blue line).

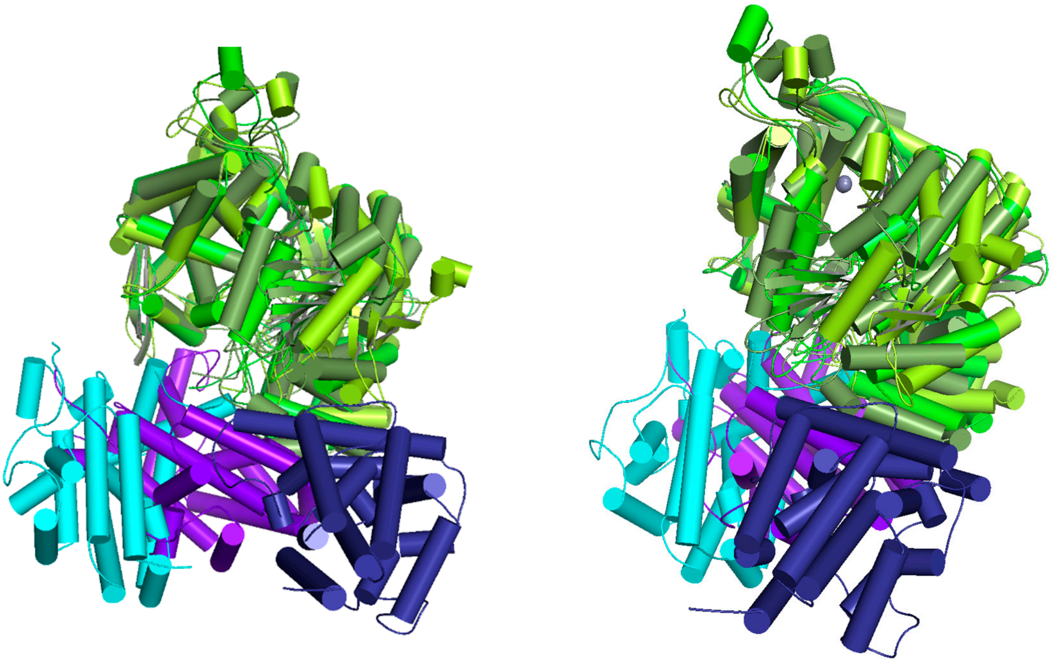

Figure S13. Superposition of the structures of the complexes from the range of lowest MMGBSA energies. Two different views are shown. Model 1 DPP3 – forest green, SH2D3C – dark blue; model 3 – DPP3 lemon green, SH2D3C – purple; model 4 – DPP3 light green, SH2D3C – cyan.

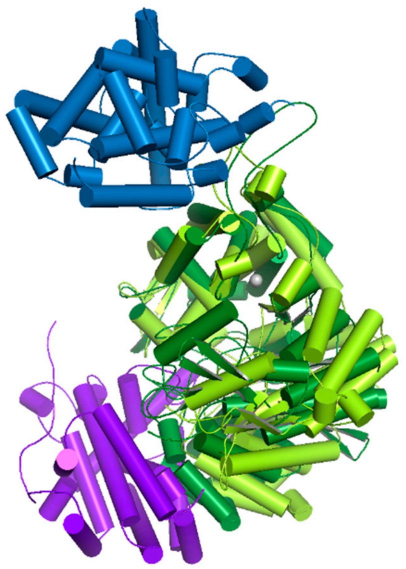

Figure S14. Superposition of the structures of the complexes from the range of the lowest MMGBSA energies obtained by simulation of models 3 and 5. Model 5: DPP3 – forest green, SH2D3C – dark blue; model 3: DPP3 – lemon green, SH2D3C – purple.

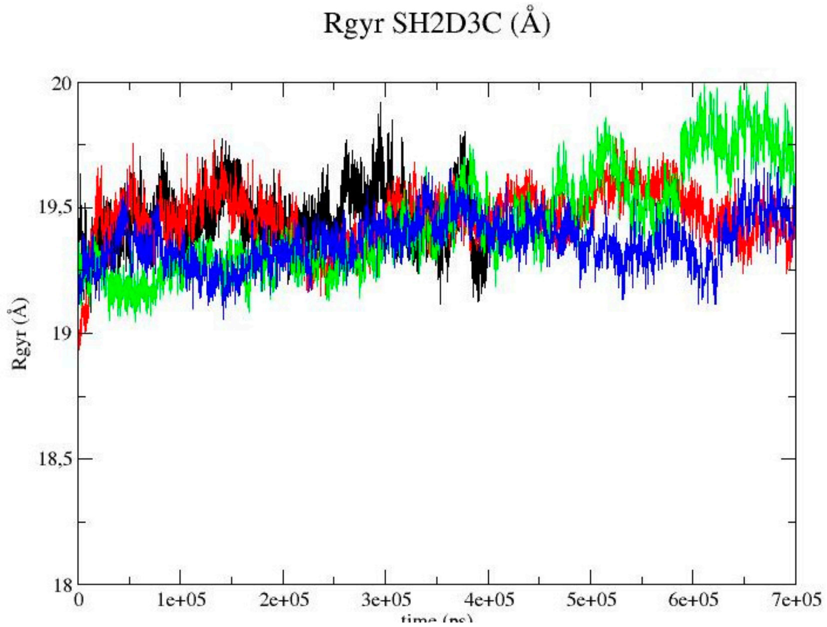

Figure S15. Rgyr of SH2D3C in different models (DPP3 – SH2D3C complex) during MD simulations (model 1 black, model 3 red, model 4 green, model 5 blue line).

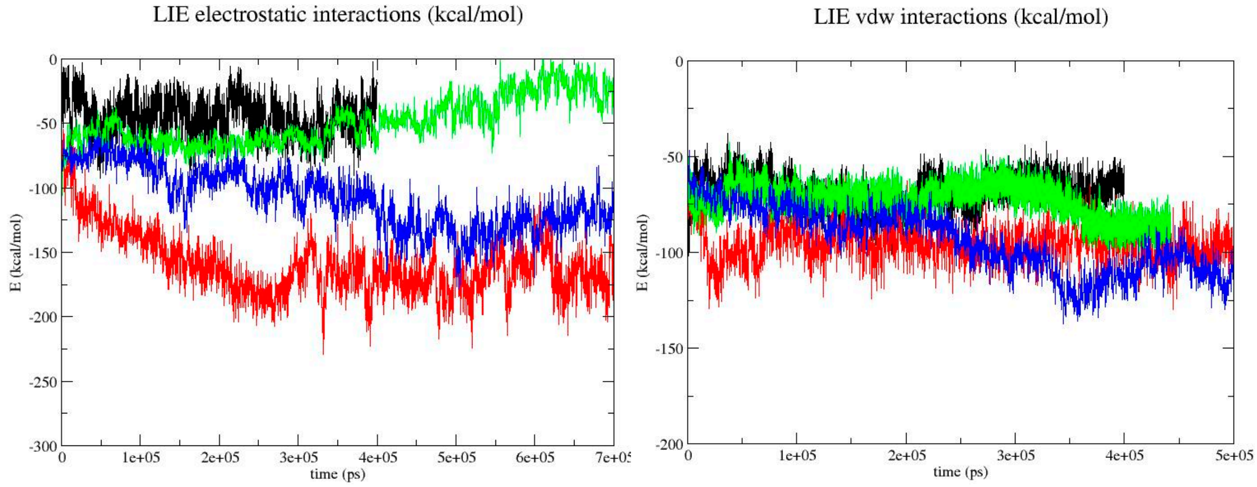

Figure S16. Electrostatic (left) and vdw (right) energies calculated by LIE method, for different models (DPP3 – SH2D3C complex) during MD simulations (model 1 black, model 3 red, model 4 green, model 5 blue line).

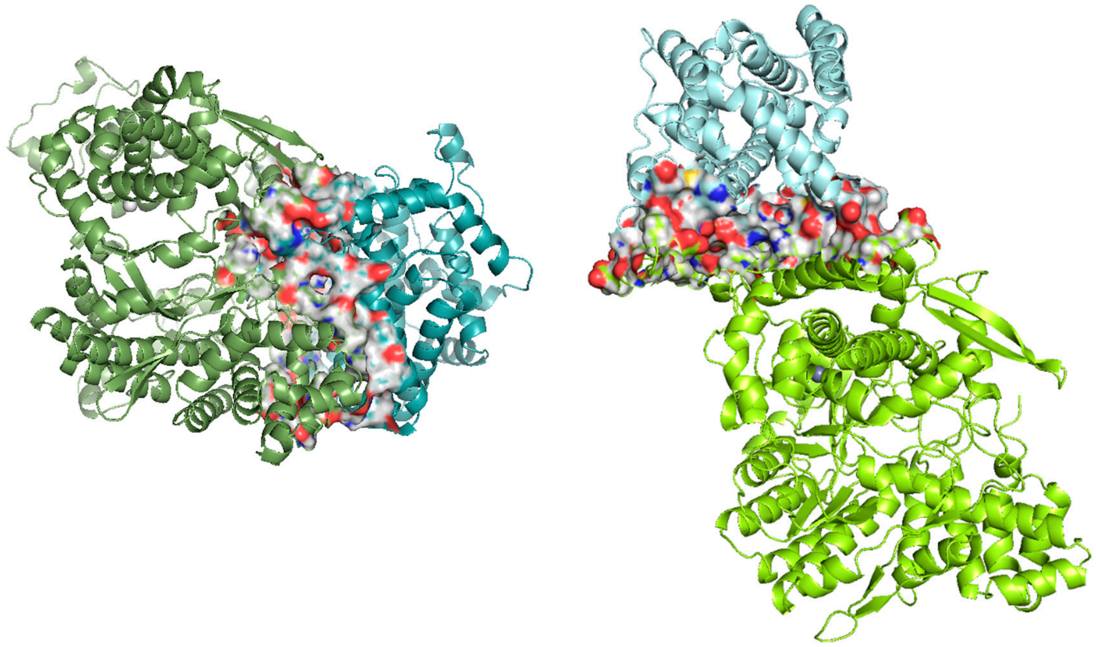

a) b)

Figure S17. Structure of the models for which MMGBSA energies were low with shown interface between DPP3 and SH2D3C: a) model 3, and b) model 5.

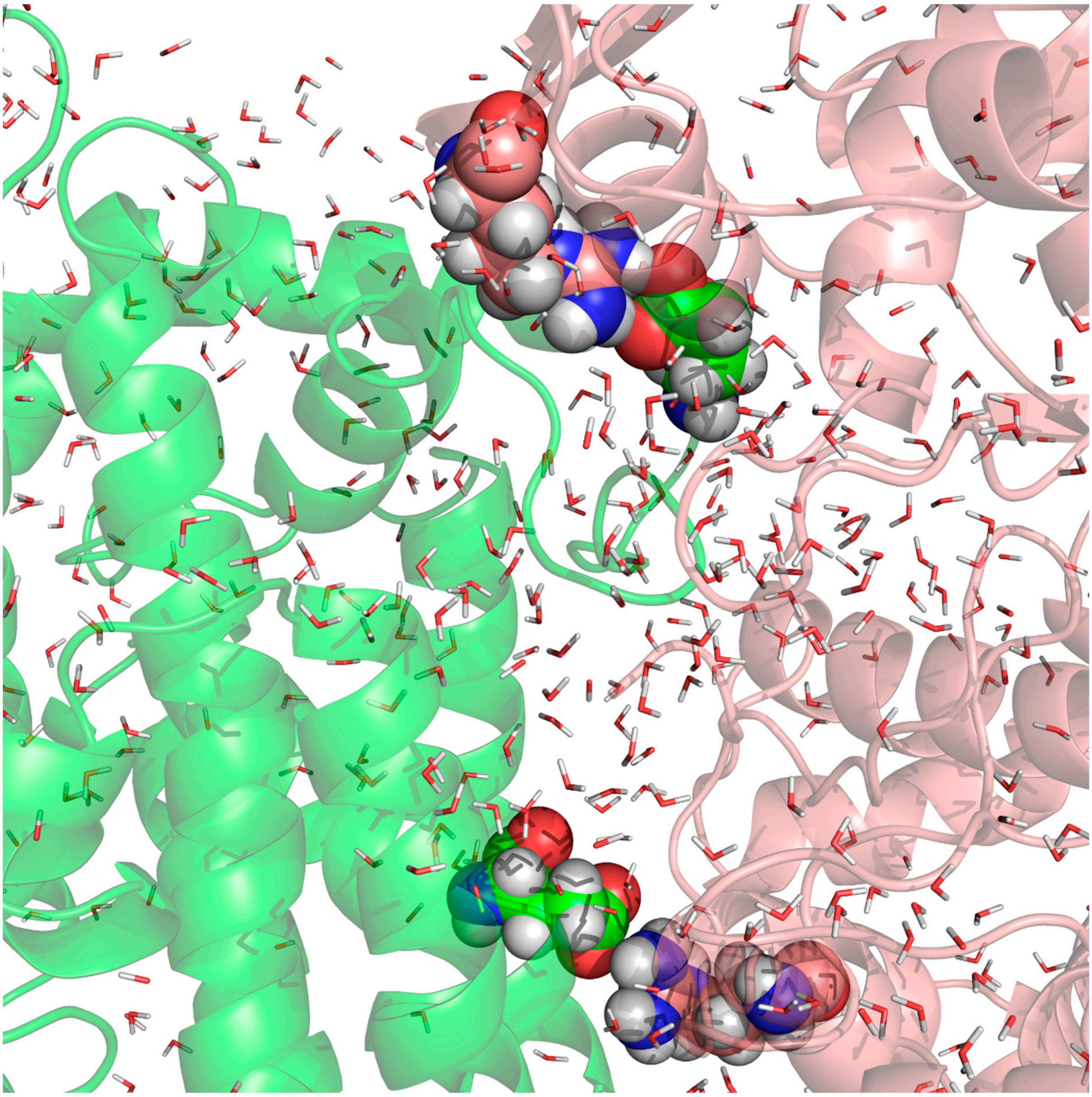

Figure S18. Water molecules accommodated between DPP3 (purple) and SH2D3C (green) in model 3. Only the water molecules within 3 Å of the protein surface are shown. The strongest hydrogen bonds between the guanidino group of R598 of DPP III and the carboxyl group of E613 of SH2D3C and between the guanidino group of R125 of DPP III and the carboxyl group of E562 of SH2D3C are shown in spherical representation.

Table S2. Solvation component of MMGBSA energies for models 3 and and 5 for the period of 200 ns of MD simulations.

Time (ns)	Model 3	Model 5
310	466.8015	351.1687
320	427.4329	369.8487
330	474.0169	363.5811

340	501.5235	457.9127
350	461.5402	432.5074
360	493.6488	404.8261
370	478.23	393.6198
380	496.7344	354.7116
390	530.615	391.8908
400	515.7376	409.0791
410	469.9923	458.9901
420	480.4632	469.3139
430	499.3576	519.8886
440	540.8209	530.308
450	513.8672	485.2719
460	521.2531	493.318
470	535.9191	499.5316
480	500.6476	449.0087
480	609.6751	464.6251
490	564.4617	533.7824
500	518.3735	351.1687
**Average (300-500)ns**	**504.8**	**441.7**

Table S3. Population of intermolecular hydrogen bonds during MD simulations. Only bonds with a population greater than 10% are shown. Data are given for simulation time of 400 ns for model 1 and for 500 ns for models 3, 4, and 5.

Model 1		Model 3		Model 4		Model 5	
Residues (DPP3-SH2D3C)	(%)	Residues (DPP3-SH2D3C)	(%)	Residues (DPP3-SH2D3C)	(%)	Residues (DPP3-SH2D3C)	(%)
E139-R461	39	T4-E607	81	T4-C588	37	E465-S669	32
T146-R627	114	Q5-E607	38	T4-G610	10	N478-V387	15
R157-E617	55	N10-E574	22	T4-E607	17	E483-R473	38
E710-R461	51	D11-T612	52	Q5-G610	20	Q484-Q474	78
T720-E624	12	D11-E613	57	Q5-S606	25	Q486-S675	60
W726-E617	18	D11-H614	23	Y6-G610	67	Q486-Q676	35
		D82-K563	21	Y6-T612	12	R620-C598	16
		E87-K576	82	L7-T612	20	R620-D599	50
		E87-K570	78	V115-T612	72	R620-E607	77
		D111- E604	78	N117-T612	47	R623-S600	18
		T112-E604	143	S595-R627	26	R623-D599	19
		N117-E574	70	R598-D599	100	R624-C598	69
		E121-R551	81	R598-H622	13	R624-D599	16
		E121-K566	21			R624-A601	20
		R125-E562	207			R624-E607	225
		R157-V698	14			D633-R678	30
		R159-D684	193				
		K165-E681	72				
		E166-K685	82				
		E422-V616	26				
		K423-E613	76				
		R598-E613	203				
		D660-S600	41				
		D660-A601	26				
		K666-P603	56				
		K666-E604	12				

Table S4. Primers used for cloning and mutagenesis

Primer	Sequence	Purpose
DPP3-TO.HA-XhoI-F	ctaCTCGAGcaATGGCGGACACCCAG	cloning of DPP3 in pcDNA4.TO.HA
DPP3-TO.HA-Xho-R	cgtaCTCGAGTTAAGCTTGGCCAGATGG	
DPP3-E451A-F	GGTGGGCCTGCACGCGCTGCTGGGCCATG	DPP3-E451A mutagenesis
DPP3-E451A-R	CATGGCCCAGCAGCGCGTGCAGGCCCACC	
FLAG-SH2D3C.FOR	cgactctagaggatccATGACAGAGGGGACCAAGAAG	cloning of SH2D3C-isoform 1 in pFLAG-CMV2
FLAG-SH2D3C.REV	atgccacccgggatccCaGCTCGCTGGAGCGG	
FLAG-SH2D3C-iso2.FOR	cgactctagaggatccATGACTGCTGTGGGCCG	cloning of SH2D3C-isoform 2 in pFLAG-CMV2
FLAG-SH2D3C.REV	atgccacccgggatccCaGCTCGCTGGAGCGG	
FLAG-SH2D3C-iso3.FOR	cgactctagaggatccATGAAGCGGCGCAGC	cloning of SH2D3C-isoform 3 in pFLAG-CMV2
FLAG-SH2D3C.REV	atgccacccgggatccCaGCTCGCTGGAGCGG	
DPP3-XhoI-F1	atttCTCGAGTTATGGCGGACACCCAGTAC	cloning of DPP3 in pEGFP-C1
DPP3-PstI-R1	ttaCTGCAGTCAAGCTTGGCCAGATGG	
KEAP1-XhoI-F	cgaCTCGAGCTATGCAGCCAGATCC	cloning of KEAP1 in pmCherry-C1

KEAP1-BamHI-R	cagGGATCCTCAACAGGTACAGTTC	
SH2D3C-N-mCherry-C1.FOR	CTAGCGCTACCGGTCATGACAGAGGGGACCAAGAAGAC	cloning of SH2D3C-isoform 1 in pmCherry-C1
SH2D3C-N-mCherry-C1.REV	GCTCACCATGGTGGCCaGCTCGCTGGAGCGG	
pcDNA3.1.FOR	GCGGCCGCTCGAGTCTAG	amplification of pcDNA
pcDNA3.1.REV	GGTGGCAAGCTTAAGTTTAAACGC	
pcDNA3.1-VenusfN.FOR	CTTAAGCTTGCCACCgataccggtCGGAGTATAGCC	cloning of VenusfN in pcDNA3.1
pcDNA3.1-VenusfN.REV	GACTCGAGCGGCCGCctatagggagagagctatgacgtcg	
pcDNA3.1-Venus-fC.FOR	CTTAAGCTTGCCACCatggataccggtCGGAGTATAGC	cloning of VenusfC in pcDNA3.1
pcDNA3.1-Venus-fC.REV	GACTCGAGCGGCCGCagctatgacgtcgcatgc	
pcDNA3.1-CfN.FOR	gataccggtCGGAGTATAGCCAC	amplification of pcDNA3.1-CfN vector for In-Fusion cloning
pcDNA3.1-CfN.REV	GGTGGCAAGCTTAAGTTTAAACGC	
pcDNA3.1-DPP3-CfN.FOR	CTTAAGCTTGCCACCATGGCGGACACCCAGT	cloning of DPP3 in pcDNA3.1-CfN
pcDNA3.1-DPP3-CfN.REV	ACTCCGaccggtatcAGCTTGGCCAGATGGG	
pcDNA3.1-CfC.FOR	atggataccggtCGGAGTATAGC	amplification of pcDNA3.1-CfN vector for In-Fusion cloning
pcDNA3.1-CfC.REV	GGTGGCAAGCTTAAGTTTAAACGC	
pcDNA3.1-SH2D3C-CfC.FOR	CTTAAGCTTGCCACCATGACAGAGGGGACCAAG	cloning of SH2D3C-isoform 1 in pcDNA3.1-CfC
pcDNA3.1-SH2D3C-CfC.REV	CCGaccggtatccatCaGCTCGCTGGAGC	
pcDNA3.1-NfC.FOR	TAGAGGGCCCGTTTAAACCC	amplification of pcDNA3.1-NfC vector for In-Fusion cloning
pcDNA3.1-NfC.REV	GACTCGAGCGGCCGCag	
pcDNA3.1-NfC-SH2D3C.FOR	GCGGCCGCTCGAGTCATGACAGAGGGGACCAAGAAGAC	cloning of SH2D3C-isoform 1 in pcDNA3.1-NfC
pcDNA3.1-NfC-SH2D3C.REV	TAAACGGGCCCTCTACaGCTCGCTGGAGCGG	
pET15b.FOR	catatggctgccgcgcgg	amplification of pET15b vector for In-Fusion cloning
pET15b.REV	TGAggatccggctgctaacaaag	
pET15b-RRas27-196.FOR	cgcggcagccatatgagcgagacgcataagttgg	cloning of RRAS in pET15b
pET15b-RRas27-196.REV	gcagccggatccTCAttgttcttggtacttacgaaccg	

1. Mace, P.D.; Wallez, Y.; Dobaczewska, M.K.; Lee, J.J.; Robinson, H.; Pasquale, E.B.; Riedl, S.J. NSP-Cas protein structures reveal a promiscuous interaction module in cell signaling. *Nat. Struct. Mol. Biol.* **2011**, *18*, 1381–1387, doi:10.1038/nsmb.2152.
